# The role of the clinical laboratory in the study of mesalazine-induced nephrolithiasis and crystalluria

**DOI:** 10.1515/almed-2025-0164

**Published:** 2025-11-10

**Authors:** Ana Isabel Balbuena, Noelia Poveda, Araceli López, María José Ferri

**Affiliations:** Hospital General de Elda Virgen de la Salud, Alicante, Spain; Department of Clinical Biochemistry, Hospital General Universitario Virgen de la Salud, Elda, Alicante, Spain; Department of Clinical Biochemistry, Hospital General Universitario Doctor Balmis, Alicante, Spain; Commission of Nephrology of the Spanish Society of Laboratory Medicine, Barcelona, Spain

**Keywords:** crystalluria, renal lithiasis, mesalazine

## Abstract

**Objectives:**

The development of urinary lithiasis is a relatively frequent complication in patients with inflammatory bowel disease (IBD). A multiplicity of factors – including medication – may induce the formation of renal stones and cause complications of variable severity.

**Case presentation:**

We report a six-case series of patients with IBD treated with mesalazine who developed renoureteral colic. Spontaneous stone expulsion occurred in three patients, with infrared spectroscopy (IRS) confirming a composition of 100 % mesalazine. In the other three cases, the presence of crystalluria was confirmed in urine sediment during the renal colic. In two of these patients, IRS analysis of dried urine sediment confirmed a mesalazine composition.

**Conclusions:**

The scarce literature available on the association between the use of mesalazine and increased risk of lithiasis fails to provide frequency data. Only a previous case on mesalazine crystalluria had been reported in the literature so far, probably due to the difficulty detecting this complication. This case series emphasizes the role of urine sediment analysis in identifying crystalluria and preventing major complications during the follow-up of patients receiving mesalazine therapy.

## Introduction

Patients with inflammatory bowel disease (IBD) are at higher risk of developing renal stones, with incidence in this population doubling that of the healthy population. Renal stones in IBD patients are associated with a higher morbidity [1]. The pathogenesis of renal lithiasis is multifactorial, including inflammation and malabsorption, having deleterious effects on hydration and electrolyte balance. Physicians should be aware of the association between mesalazine and nephrolithiasis to ensure early diagnosis and prevent complications, including urinary tract infections, sepsis, acute kidney failure, even leading to the development of interstitial nephritis and chronic kidney disease [2], 3]. In these patients, lithiasis are most frequently composed of calcium oxalate and uric acid. For this reason, when these patients present with a renal colic, a pharmacological composition is not suspected.

The first stage in the development of kidney stones is the formation of crystals. Drug-related crystalluria is a rare finding in urine sediment that may induce acute kidney failure. Often, the composition of these crystals is not identified. The estimated incidence of drug-induced urolithiasis is 1–2 % for the general population. Causative factors include metabolic effects on the kidneys or, in the case of poorly water-soluble drugs with high renal excretion rates, to the precipitation of the drug or its metabolites in urinary ducts [4], [5], [6], [7]. Differentiating these lithiasis may be challenging. Among the different methods available, drug-induced lithiasis cannot be identified by biochemistry testing. In turn, imaging techniques (X-ray, CT scan, ultrasonography) can provide guidance on stone composition based on their morphological characteristics, but their exact composition cannot be determined by these techniques. Infrared spectroscopy (IRS) is the technique recommended by the European Association of Urology. This technique enables the exact identification of lithogenic drugs by comparing their spectra with other known spectra. However, this technique is not available in all hospitals.

If the spectrum is not recorded in the database, non-conclusive results may be obtained, which may contribute to underdiagnosis.

Mesalazine (5-aminosalicylic acid, 5-ASA) is an anti-inflammatory drug used as first-line therapy for inflammatory bowel disease. This drug is administered either orally or rectally at a recommended dose of 2 g/day for adults. When administered orally, 60 % of mesalazine is absorbed by the small bowel, thereafter it is metabolized in the liver and other tissues and primarily excreted via the kidneys. Adverse events (AEs) reported in the mesalazine SmPC by the Spanish Agency for Medicines and Medical Devices (AEMPS) include pericarditis, pancreatitis, hepatitis, nephrotoxicity, alveolitis, blood dyscrasias, along with renal function disorders such as acute and chronic interstitial nephritis, nephrotic syndrome and renal failure. Other very rare AEs (<1:10.000) or AEs with an unknown frequency or whose frequency cannot be estimated include nephrolithiasis and urine decoloration [8]. Renal colic has been reported in the literature as an AE of mesalazine, with a few published cases of mesalazine-induced nephrolithiasis [1], 4], 5]; however, confirmation of crystalluria was obtained in only one case [4]. In addition, a case of hematuria as an AE of mesalazine has also been described [9].

## Case presentation

We present three cases of renal lithiasis and another three cases of crystalluria in patients with IBD treated with mesalazine who suffered one or more episodes of renal colic as a complication of the treatment.

The stones and crystals in urine sediment were analyzed by IRS (Spectrum Two; Perkin Elmer; (Waltham, Massachusetts, USA)) in five of the six cases.

**Figure 1:**
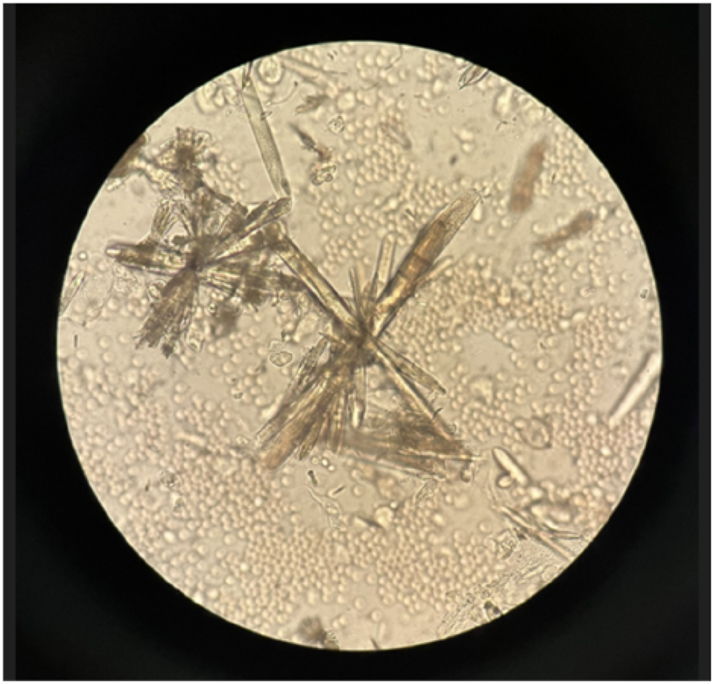
Mesalazine crystals in a urine sediment. Bright-field observation at 400× using an Olympus optical microscope CX41.

**Figure 2:**
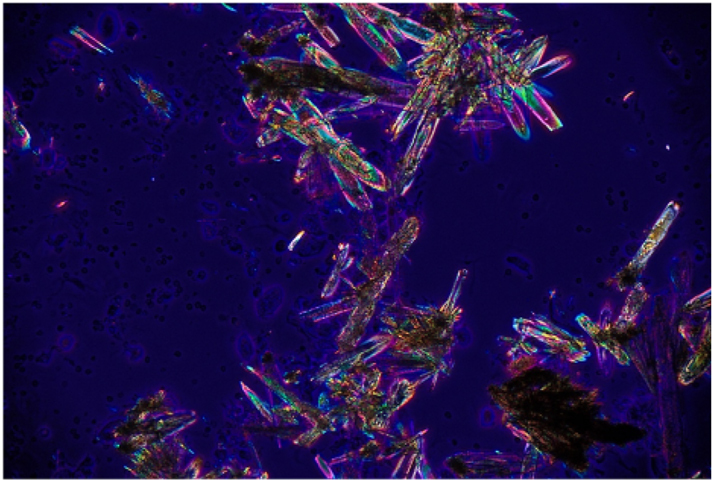
Mesalazine crystals in a urine sediment. Examination under polarized light at 400× magnification with an Olympus CX41 microscope.

**Figure 3:**
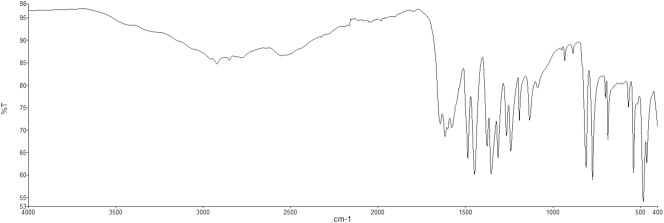
Infrared spectroscopy of a urine sediment with mesalazine crystalluria. Theoretical spectrum show in black, patient spectrum in red.

Of the six cases presented, five had received a diagnosis of ulcerative colitis and one of Crohn’s disease (Table 1). Three patients were male and three female, with ages ranging from 21 to 43 years. All patients were receiving mesalazine orally at a dose of 4 g a day and had a history of at least one episode of renoureteral colic (RC) diagnosed between May 2022 and October 2024. The majority of patients had suffered multiple episodes of RC since the initiation of treatment but had no history of RC prior to treatment. Two patients suffered more than 10 documented episodes, whereas another patient had five documented episodes, although more than 10 episodes were reported on his medical record in a year. The time from initiation of mesalazine therapy to the occurrence of the first documented RC was very variable, ranging from less than one month (two cases) to 8 years (one case). Two patients developed hydronephrosis as a complication during a RC episode. Three of the six cases achieved spontaneous stone expulsion. Infrared spectroscopy (IRS) of these lithiasis confirmed a 100 % mesalazine composition. The other three cases could not expulse stone, but the presence of crystalluria was confirmed by urine sediment microscopy concurrently to a RC episode (Figures 1 and 2). In the first case observed, crystal composition could not be determined. In the other two cases, IRS analysis of previously dried urine sediment confirmed its mesalazine composition (Figure 3). Oral mesalazine therapy was discontinued in two of the patients who developed lithiasis, whereas the dose was downtitrated to 2 g/day in another case. One of the patients discontinued treatment on his own initiative.

**Table 1:** Presence of lithiasis/crystalluria in patients with IBD treated with mesalazine who developed one or more episodes of RC.

Case	1	2	3	4	5	6
Sex	Female	Female	Male	Female	Male	Male
Age	21	42	43	38	25	32
Diagnosis	Crohn’s disease	Ulcerative colitis	Ulcerative colitis	Ulcerative colitis	Ulcerative colitis	Ulcerative colitis
Mesalazine dose	4 g/day	4 g/day	4 g/day	4 g/day	4 g/day	4 g/day
Duration of treatment	5 years	6 years	4 years	11 years	4 months	10 years
RC episodes	13	1	4	5	1	12
Time from treatment initiation to first RC	<1 month	5 years	7 months	3 years	<1 month	8 years
Lithiasis	No	Yes	Yes	No	No	Yes
Crystalluria	Yes	No	No	Yes	Yes	No
IRS	No	Yes	Yes	Yes	Yes	Yes

RC, renal colic; IRS, infrared spectroscopy.

## Discussion

Mesalazine-induced nephrolithiasis is an adverse reaction described by the AEMPS; however, the frequency of this complication has not yet been established. Most of the cases have been reported in the last 3 years at first to sixth month of treatment, with a comparable incidence in men and women [10]. The number of case series reporting lithiasis composition is very limited. Beyond our case series, only a case of mesalazine crystalluria had been reported so far, in the case series published by Chebion et al. in 2021 [4].

Crystalluria is the previous step to lithiasis. The presence of large crystals, aggregates, and persistent crystalluria are risk factors for lithiasis [11]. In drug-induced crystalluria, crystal composition is not consistently identified, as the technique may not be available. Additionally, crystalluria may remain unnoticed in the absence of urinary symptoms or when urine dipstick findings do not warrant sediment analysis by optical microscopy. The identification of crystals in urine sediment is a relevant finding in patients receiving mesalazine, as it indicates a higher risk of developing lithiasis. However, the volume of urine samples tested daily, added to the unavailability of supplementary clinical details in most cases, may result in crystalluria remaining undetected. To date, an automated standard operating procedure is not available in clinical laboratories for the detection of infrequent crystals in urine. Hence, crystalluria is most frequently identified when patients are referred for urine sediment testing for other reasons or when the test is explicitly ordered.

## Highlights

– Mesalazine therapy may be associated with renoureteral colic, the presence of crystalluria and the formation of urinary lithiasis.
– The analysis of urine sediment in these patients is a valuable tool for the etiologic diagnosis of this type or renoureteral colics.
– Follow-up of these patients should incorporate urine sediment analysis to facilitate early detection of complications and, when necessary, adjustment of treatment or dosage.
– Clinicians should communicate directly with laboratory specialists when diagnostic suspicion arises, providing relevant clinical information and explicitly requesting targeted crystal analysis.
